# Childhood DNA methylation as a marker of early life rapid weight gain and subsequent overweight

**DOI:** 10.1186/s13148-020-00952-z

**Published:** 2021-01-12

**Authors:** N. Robinson, H. Brown, Elie Antoun, Keith M. Godfrey, Mark A. Hanson, Karen A. Lillycrop, Sarah R. Crozier, Robert Murray, M. S. Pearce, C. L. Relton, V. Albani, J. A. McKay

**Affiliations:** 1 Population Health Sciences, Newcastle University Medical School, Newcastle University, Newcastle upon Tyne, UK; 2 Institute of Developmental Sciences, Biological Sciences and NIHR Southampton Biomedical Research Centre, University of Southampton, Southampton, UK; 3 MRC Lifecourse Epidemiology Unit and NIHR Southampton Biomedical Research Centre, University of Southampton and University Hospital Southampton NHS Foundation Trust, Southampton, UK; 4 MRC Integrative Epidemiology Unit, Population Health Sciences, Bristol Medical School, University of Bristol, Bristol, UK; 5 Department of Applied Sciences, Northumbria University, Newcastle upon Tyne, UK

**Keywords:** Rapid weight gain, Conditional weight gain, Epigenetics, EWAS, DNA methylation, DOHAD, ALSPAC, SWS

## Abstract

**Background:**

High early postnatal weight gain has been associated with childhood adiposity; however, the mechanism remains unknown. DNA methylation is a hypothesised mechanism linking early life exposures and subsequent disease. However, epigenetic changes associated with high early weight gain have not previously been investigated. Our aim was to investigate the associations between early weight gain, peripheral blood DNA methylation, and subsequent overweight/obese. Data from the UK Avon Longitudinal study of Parents and Children (ALSPAC) cohort were used to estimate associations between early postnatal weight gain and epigenome-wide DNA CpG site methylation (Illumina 450 K Methylation Beadchip) in blood in childhood (*n* = 125) and late adolescence (*n* = 96). High weight gain in the first year (a change in weight z‐scores > 0.67), both unconditional (rapid weight gain) and conditional on birthweight (rapid thrive), was related to individual CpG site methylation and across regions using the meffil pipeline, with and without adjustment for cell type proportions, and with 5% false discovery rate correction. Variation in methylation at high weight gain-associated CpG sites was then examined with regard to body composition measures in childhood and adolescence. Replication of the differentially methylated CpG sites was sought using whole-blood DNA samples from 104 children from the UK Southampton Women’s Survey.

**Results:**

Rapid infant weight gain was associated with small (+ 1% change) increases in childhood methylation (age 7) for two distinct CpG sites (cg01379158 (*NT5M*) and cg11531579 (*CHFR*)). Childhood methylation at one of these CpGs (cg11531579) was also higher in those who experienced rapid weight gain and were subsequently overweight/obese in adolescence (age 17). Rapid weight gain was not associated with differential DNA methylation in adolescence. Childhood methylation at the cg11531579 site was also suggestively associated with rapid weight gain in the replication cohort.

**Conclusions:**

This study identified associations between rapid weight gain in infancy and small increases in childhood methylation at two CpG sites, one of which was replicated and was also associated with subsequent overweight/obese. It will be important to determine whether loci are markers of early rapid weight gain across different, larger populations. The mechanistic relevance of these differentially methylated sites requires further investigation.

## Background

Children are becoming obese at younger ages [1], suggesting that factors in early life may play a role in obesity development. The developmental origins of health and disease (DOHaD) hypothesis proposes that early life environmental exposures have the potential to modify the risk of later-life diseases, such as obesity [2]. Rapid weight gain (RWG) is an early life factor that has been consistently associated with childhood adiposity both dependently and independently of birthweight [3–5]. Weight gain in the first year specifically (opposed to change in weight over periods greater or less than 1 year) has been found to be most predictive of childhood obesity [6], suggesting this is a critical period.

Given the responsiveness to environmental stimuli, the capacity to alter gene expression and their stability over time, epigenetic changes are a proposed mechanism underlying the DOHaD hypothesis. Through programming effects, epigenetic marks laid down at an early developmental stage could elicit effects at a later stage [7]. DNA methylation (DNAm) is the most stable and widely studied epigenetic modification and is a key mechanism regulating gene expression. DNAm involves the covalent addition of a methyl group to cytosine residues adjacent to guanine in DNA (CpG sites) and is associated with changes in gene transcription [8]. If early life factors lead to stable changes in DNAm, these changes could be used as biomarkers and to identify individuals who may benefit from intervention prior to disease onset.

BMI has been associated with variation in DNAm from birth to adulthood [9–12]. Epigenome-Wide Association Studies (EWAS) are a comprehensive approach to identify epigenetic variation associated with a biological trait or exposure [13, 14]. In EWAS, other early life risk factors for childhood obesity such as birthweight and maternal BMI have been associated with variation in DNAm [15, 16]. To our knowledge, there have been no EWAS to date on early life rapid growth and DNAm.

Our first aim was to identify DNAm changes associated with early life growth. In this study we hypothesised that early life rapid growth is associated with DNAm changes. Using epigenome-wide DNAm array data from the Avon Longitudinal Study of Parents and Children (ALSPAC) cohort, we investigated if early life rapid growth is associated with variation in childhood methylation, and if methylation changes persist into adolescence. As catch up growth is more likely in low birthweight infants, we investigated rapid growth both adjusted (rapid thrive, RT) [17] and unadjusted for birthweight (rapid weight gain, RWG) [4]. We also examined differential methylation in a subset of known BMI-associated CpG loci [12] with the aim of identifying differentially methylated loci more likely to be related to body composition, and by analysing fewer loci, to offset the multiple comparison problem often associated with null findings in EWAS.

An important consideration is that not all children with rapid infancy weight gain will have increased adiposity in childhood [18], and previous studies have highlighted the necessity to distinguish infants at greatest risk of overweight/obesity. Therefore, we also aimed to explore if differential methylation was associated with later life BMI and overweight/obesity in those who experienced early rapid weight gain to determine potential risk markers. Finally, we sought replication in an independent cohort, the UK Southampton Women’s survey.

## Methods

### Cohort

We performed our initial analysis in the Avon Longitudinal Study of Parents and Children (ALSPAC) cohort, which has detailed early life, anthropometric and epigenome-wide DNAm data at multiple time points. This ongoing longitudinal birth cohort, based in Bristol, England (UK), initially invited pregnant women resident in Avon, UK, with expected dates of delivery 1 April 1991 to 31 December 1992 [19, 20]. There were 14,541 initial pregnancies enrolled (for these at least one questionnaire has been returned or a “Children in Focus” clinic had been attended by 19/07/99), with a total of 14,676 foetuses, resulting in 14,062 live births and 13,988 children who were alive at 1 year of age.

The cohort has had extensive questionnaires as well as clinical assessments (including measures of height & weight) throughout childhood. The Accessible Resource for Integrated Epigenomic Studies (ARIES) is a subset of the ALSPAC cohort [21], for which epigenome-wide DNAm analysis was carried out for 1,018 mother and child pairs. Ethical approval for the study was obtained from the ALSPAC Ethics and Law Committee and local research ethics committees. The study website contains details of all the data that are available through a fully searchable data dictionary accessible at www.bris.ac.uk/alspac/researchers/data-access/data-dictionary/.

### Early life data

**Fig. 1:**
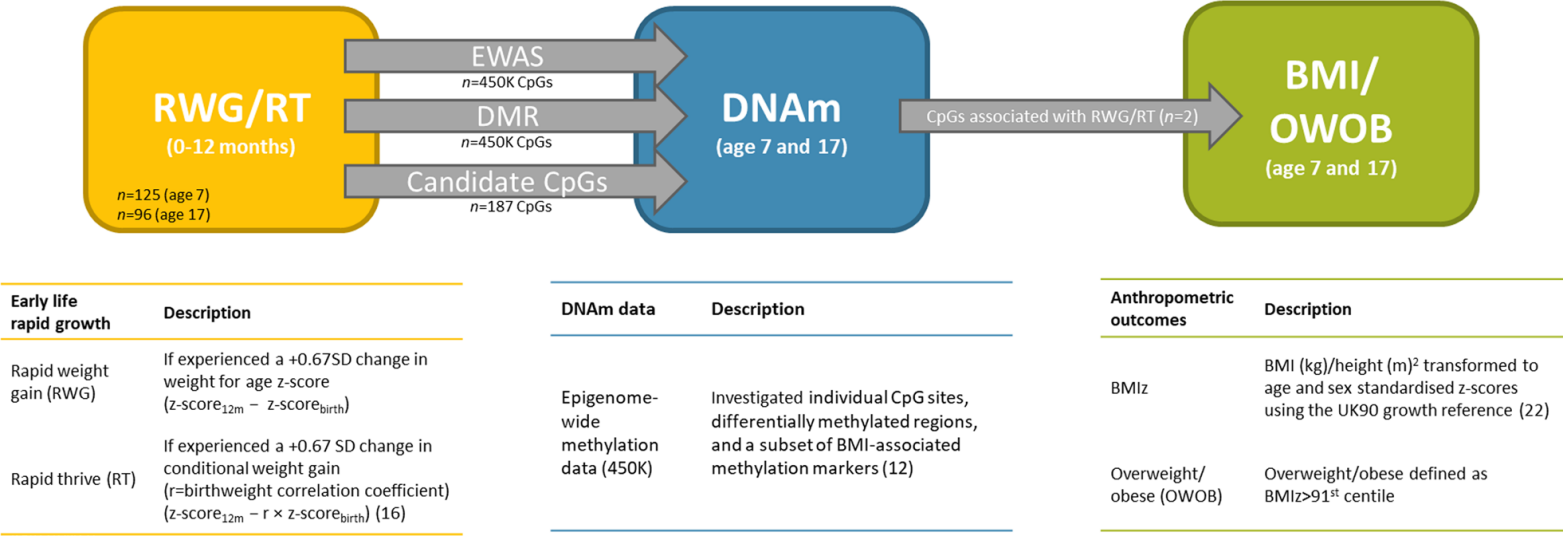
Description and measurement of rapid infant weight gain exposures, and epigenome-wide DNA methylation and anthropometric outcomes, and an overview of the analysis. Participants were measured at around age 7 (mean 7.4 (SD 0.10) years) and 17 (mean 17.7 (SD 0.31) years). RWG, rapid weight gain; RT, rapid thrive; BMIz, BMI z-scores; OWOB, overweight/obese; DNAm, DNA methylation; EWAS, Epigenome-wide association study; UK90, the British 1990 growth reference

Birthweight and gestational age were taken from medical records. At 12 months, infants were weighed using the Seca 724 (or Seca 835 for children who could only be weighed with a parent). Birthweight and weight-for-age [12 months] z-scores were calculated using the British 1990 growth reference [22] and were used to determine the rapid growth variables: RWG and RT. Whilst birthweight is an important factor in childhood obesity [23], it has previously been examined in the ALSPAC cohort [15, 24]. However, in order to differentiate the effects of birthweight on early life rapid growth, both RWG (not conditional on birthweight) and RT (conditional on birthweight) in the first year were examined (Fig. 1). Conditional weight gain (also known as thrive index) accounts for normal catch-up growth from low birthweight as a linear measure of weight gain adjusted for regression to the mean [17]. Rapid thrive was determined as *z*-score_12m_ − *r* × *z*-score_birth_ [17], where *r* is the cohort regression coefficient (*r* = 0.35) of birthweight-z on weight-z (12 months). RWG is most often defined as *a* > + 0.67 standard deviation change in weight-for-age z-score, equivalent to crossing a growth centile band on a standard child growth chart [4]. Both RWG and RT were analysed as a dichotomised variables of *a* > + 0.67 standard deviation change (Fig. 1).

### Anthropometric data

Anthropometric measures at (approximately) age 7 and 17 were also analysed as outcomes in analyses. At age 7, height was measured to the nearest millimetre without shoes or socks using a Holtain stadiometer (Holtain Ltd, Crymych, Pembs, UK), whilst weight was measured using Tanita THF 300GS body fat analyser and weighing scales (Tanita UK Ltd, Yewsley, Middlesex, UK). At age 17, height was measured with a Harpenden stadiometer to the nearest mm, and weight using the Tanita Body Fat Analyser (Model TBF 401A) to the nearest 50 g.

BMIz and overweight/obese were examined as outcomes at both time points. Body mass index (BMI) was calculated as weight (kg) divided by height (m) squared and was transformed to age and sex standardised BMI z-scores using the British 1990 growth reference with the zanthro program in Stata [25]. Clinical cut-offs were used to determine weight categories, whereby healthy weight was between the 2nd and 91st centiles, and overweight/obese as greater than the 91st centile [26].

### Epigenetic data

ALSPAC collected (peripheral) blood at ages 7 and 17 and DNA was extracted. Epigenome-wide DNAm at specific CpG sites was measured for ~ 1000 individuals using the Infinium® HumanMethylation450K BeadChip assay (Illumina, Inc., CA, USA). DNAm data were pre-processed, including background correction and subset quantile normalization using the pipeline described by Touleimat and Tost [27] (further details in the ARIES cohort profile [21]). Estimation of white blood cell counts (CD8T cells, CD4T cells, Natural Killer, B cells, Monocytes and Granulocytes) was done using the Houseman algorithm [28]. Cross-reactive and polymorphic probes identified by Chen et al. [29] and probes on sex chromosomes were removed prior to downstream analysis (*n* = 453,723 probes). Due to few participants with non-Caucasian or missing ethnicity, and few non-singleton births, these were removed.

### Statistical analysis

To examine if childhood or adolescent DNAm in peripheral blood (around ages 7 and 17) was associated with early life rapid growth, three different analyses were undertaken, including: analysis of differentially methylation positions, differentially methylated regions, and differentially methylated positions in a subset of candidate loci.

For each analysis, DNAm was the outcome and the independent variable was either RWG or RT, with models adjusted for sex and age at blood collection. Cell type composition is a significant source of variation in DNAm analysis [30]; however chronic, low level inflammation is a component of the obesity phenotype; therefore, to find novel biomarkers associated with this phenotype, DNAm was investigated in models with and without adjustment for cell composition (all 6 cell types). Correction for multiple testing was applied using a false discovery rate (FDR) threshold of *p* < 0.05 [31].

First, epigenome-wide association studies (EWAS) were conducted for rapid growth (first year) and DNAm outcomes in childhood (age 7.4) and adolescence (age 17.6) in the Meffil R package [32]. Estimation of differentially methylated sites was carried out using the beta values as the outcome and rapid growth (RWG/RT) as the exposure adjusted for age and sex. Surrogate variable analysis (SVA) and independent surrogate variable analysis (ISVA) methods were utilised to control for unmodelled or unknown confounding factors (such as batch) [33, 34]. Meffil simultaneously computes unadjusted, adjusted, SVA and ISVA models, thereby allowing results to be compared [32]. In order to minimise the influence of outliers in methylation data, beta values were winsorised at the level of 5% (95th percentile cut-off).

Second, in order to detect a DNAm signature of rapid growth, differentially methylated regions (DMRs) were analysed. DMRs, which are stretches or clusters of neighbouring CpG probes, may have more of a functional effect on gene expression than individual CpG loci [35]. Additionally, if changes in DNAm are small but persistent across a region, there may be more statistical power to detect them collectively as DMRs [36]. The R package DMRcate was used for the estimation of DMRs [37], using the M-values and default settings. The surrogate variables (that were calculated using Meffil in the EWAS models) were included as covariates in the DMR models.

Finally, in order to focus on loci with anticipated associations with adiposity, a candidate gene approach was taken using a subset of CpG sites robustly associated with BMI. The candidate sites were selected from a large-scale meta-analysis EWAS which utilised data from multiple cohorts of European and Indian-Asian descent [12]. After validation, 187 CpG sites were associated with BMI. We conducted EWAS for both RWG and RT using the 187 identified CpG sites as candidates, at both time points using the Meffil R package [32].

Any significantly differentially methylated sites were analysed further to determine whether DNAm was also associated with body composition (at age 7 and 17). Using linear regression, significant CpG sites were examined with respect to childhood and adolescent BMI (dependent variable). Similarly, as differentially methylated loci were identified in the RWG EWAS, the CpG sites were also examined with regard to RT in adjusted linear models. All models were adjusted for age and sex. Differences in DNAm by phenotype (RWG, overweight/obese) were assessed using ANOVA tests between groups (with Bonferroni correction for multiple testing).

All EWAS and bioinformatic analyses were done in Rstudio version 3.3.2. The human reference genome (GRCh37/hg19 assembly) was used to determine the location and features of the gene region using the UCSC Genome Browser [38]. Recoding of the variables and statistical analysis of differentially methylated sites was done in Stata version 15.1 (StataCorp, College Station, Texas, USA).

### Replication analysis

CpG sites with FDR *P*-values < 0.05 were carried forward for replication using DNA methylation data from the children from the Southampton Women’s Survey (SWS), a similar UK-based cohort [39]. Blood methylation measures (EPIC array) and early life weight data for 104 of the SWS children (age = 11–13 years, all Caucasian) were available. Further details of the replication cohort and methylation data processing are provided in the Additional file 1. The exact same models were estimated for the differentially methylated CpG sites in childhood (age 11–13) and early life rapid growth (RWG and RT, 0–12 months) using the meffil R package with both SVA and ISVA. Models were all adjusted for child’s sex and age at DNAm measurement and were run with and without adjustment for cell type composition.

## Results

### Descriptive characteristics

Early life growth and 450 K array data were available for 125 ALSPAC children at age 7 and 96 at age 17 (Table 1). Weight at 12 months was measured in a fraction (*n* = 1,432, 9.3%) of ALSPAC children, thereby limiting the sample size for the methylation analysis. The ARIES sub-sample was mostly representative of the main study population; however, ARIES mothers were slightly older, less likely to have a manual occupation and were less likely to smoke during pregnancy [21]. At age 7, 13% of study members were overweight/obese, and 19% were at age 17 (Additional file 2: Table 1).

**Table 1:** The proportion of individuals in the study sample with RWG or RT at age 7 and 17

Age	Variable	Models adjusted for cell counts					Models not adjusted for cell counts				
		Total	No	%	Yes	%	Total	No	%	Yes	%
7	RWG	116	75	64.7	41	35.3	125	84	67.2	41	32.8
	RT	116	65	56.0	51	44.0	125	73	58.4	52	41.6
17	RWG	89	54	60.7	35	39.3	96	61	63.5	35	36.5
	RT	89	50	56.2	39	43.8	96	56	58.3	40	41.7

Presented for models adjusted or unadjusted for cell counts. RWG, rapid weight gain; RT, rapid thrive

94/125 (75%) with measures at age 7 had DNAm measures at age 17

### EWAS results

We observed associations (P_FDR_ < 0.05) between RWG and individual CpG loci at age 7. Across the adjusted models, there were 4 associations identified for RWG (P_FDR_ < 0.05) corresponding to 2 unique CpG sites (Table 2). These loci were cg01379158 (*NT5M*) and cg11531579 (*CHFR*), and both were associated with a 1% increase in methylation (P_FDR_ = 0.02) in those who had RWG. In the models without cell counts, two of the model *p* values were also below the Bonferroni *p* value threshold (1.04 × 10^–7^). There were no associations (P_FDR_ < 0.05) between RT and individual CpG loci at age 7 in the EWAS. We examined whether methylation at the RWG-associated CpG sites was also associated with RT using regression analysis; however the magnitude of the coefficients was lower and less statistically robust than for RWG (Additional file 2: Table 2). There was no evidence that RWG or RT was associated (P_FDR_ < 0.1) with differential DNAm in adolescence for the EWAS, or for the 2 CpG sites identified as differentially methylated at age 7 using linear regression (Additional file 3). In the DMR analysis, there were no overall DMRs identified; all Stouffer corrected *p* values were non-significant, suggesting a lack of consistency in the direction of the methylation changes.

**Table 2:** Associations (FDR p < 0.05) between individual CpG sites (age 7, *n* = 453,723) and the early life growth in models

Exposure	*n*	CpG name	Nearest gene	Gene region	CpG island name	Model	Coef	SE	*P*	P_FDR_
*With cell counts*										
RWG	116	cg01379158	*NT5M*	TSS200	chr17:17,206,527–17,207,306	ISVA	0.011	0.0018	2.91 × 10^–7^	0.02
*Without cell counts*										
RWG	125	cg01379158	*NT5M*	TSS200	chr17:17,206,527–17,207,306	ISVA	0.011	0.0017	1.41 × 10^–8^	0.01
RWG	125	cg11531579	*CHFR*	Island	chr12:133,484,658–133,485,739	SVA	0.011	0.0019	4.16 × 10^–8^	0.02
RWG	125	cg11531579	*CHFR*	Island	chr12:133,484,658–133,485,739	ISVA	0.011	0.0019	1.26 × 10^–7^	0.03

All associations FDR p < 0.05 are presented from the ISVA and SVA models, and the models with and without adjustment for cell types. Chr, chromosome; P_FDR_, FDR *p* value; P, unadjusted *p* value, Coef, coefficient; TSS200, transcription start site; RWG, rapid weight gain; SVA, Surrogate variable analysis, and ISVA, independent surrogate variable analysis. Bonferroni *p* value threshold = 1.04 × 10^–7^

### The candidate gene analysis

The aim of the candidate gene analyses was to select CpG loci already known to be associated with the outcome phenotype of interest (body composition). Using a smaller subset of loci as candidates has the advantage of reducing the stringent *p* value threshold when correcting for multiple tests. The candidate gene analysis utilised findings from a consortium, which integrated data from 4 discovery cohorts and replicated findings in 9 cohorts, and found 187 validated methylation markers associated with BMI [12].

The associations between the candidate CpG loci (*n* = 187) and early life rapid growth were examined using the ALSPAC methylation childhood and adolescent data; however there were no associations identified (Bonferroni *p* value > 3 × 10^–4^).

### Investigating phenotypic differences in DNAm associated with RWG

**Fig. 2:**
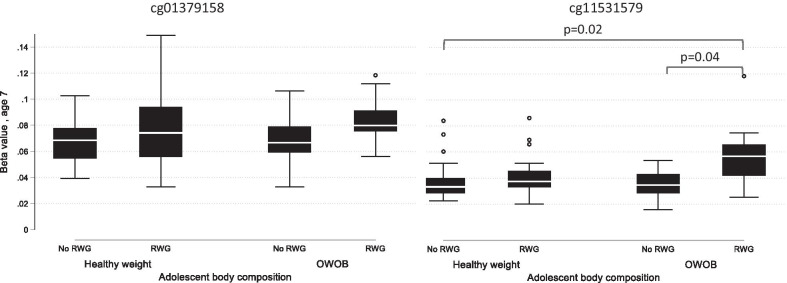
Childhood methylation and adolescent body composition. Methylation at the cg01379158 locus (left plot), and cg11531579 (right plot). There were no differences between groups for cg01379158 ANOVA (*p* > 0.05). The cg11531579 plot presents tests for significance (*p* values) from the Kruskal–Wallis test with Bonferroni correction for multiple testing. RWG, rapid weight gain; OWOB, overweight/obese

Methylation at either CpG site was not directly associated with BMIz at age 7 or 17 in regression analyses. For both CpG sites, highest methylation was in those who had RWG and were subsequently overweight/obese (compared to healthy weight), both in childhood and adolescence (Additional file 2: Table 3). At the cg11531579 site, childhood methylation was higher in those who were subsequently overweight/obese (age 17) and had RWG compared to those who did not have RWG (Fig. 2, ANOVA *p* < 0.05). However, the sample sizes for these groups were small and therefore results are inconclusive.

**Table 3:** Associations between early life rapid growth and childhood methylation in the replication cohort (SWS children age 11–13, *n* = 104)

Exposure	CpG site	Nearest gene	ISVA			SVA		
			Coef	SE	P	Coef	SE	P
*Models with cell counts*								
RWG	cg11531579	CHFR	0.0045	0.0020	0.02	0.0045	0.0021	0.04
RWG	cg01379158	NT5M	0.0004	0.0029	0.90	− 0.0022	0.0026	0.40
RT	cg11531579	CHFR	0.0022	0.0026	0.39	0.0029	0.0024	0.23
RT	cg01379158	NT5M	− 0.0002	0.0036	0.96	− 0.0030	0.0029	0.31
*Models without cell counts*								
RWG	cg11531579	CHFR	0.0005	0.0020	0.82	− 0.0002	0.0020	0.92
RWG	cg01379158	NT5M	0.0019	0.0023	0.40	0.0020	0.0023	0.40
RT	cg11531579	CHFR	− 0.0013	0.0020	0.52	− 0.0020	0.0022	0.37
RT	cg01379158	NT5M	0.0043	0.0027	0.12	0.0018	0.0026	0.48

P, unadjusted *p* value, Coef, coefficient; SE, standard error; RWG, rapid weight gain; RT, rapid thrive; SVA, Surrogate variable analysis, and ISVA, independent surrogate variable analysis

**Fig. 3:**
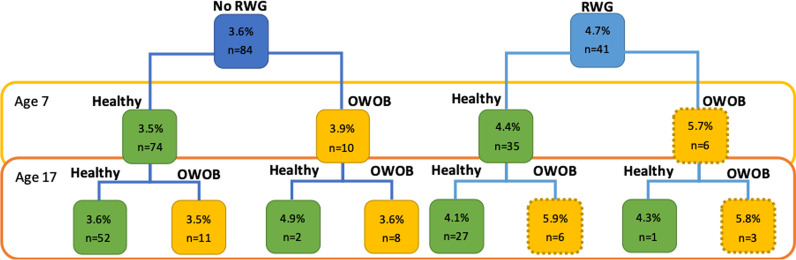
Pathways of mean methylation (cg11531579) at age 7 (%) and body composition (at ages 7 and 17). Dotted outline indicates the ‘high-risk’ individuals (i.e. those who had RWG and were subsequently overweight/obese (OWOB). Sample sizes are for those with complete data. Group sizes were small for some phenotypes.

Furthermore, those who were healthy weight at age 7 but were overweight/obese at age 17 had higher methylation at age 7 (Fig. 3), whereas those who had RWG but were a healthy weight (at either time point) had consistently lower levels of methylation. On average, methylation was lower in those who did not have RWG regardless of weight status. Although group sizes were small (*n* = 6), methylation at age 7 could have indicated future risk of overweight/obesity in the ‘high-risk’ group.

### Methylation change over time within individuals

**Fig. 4:**
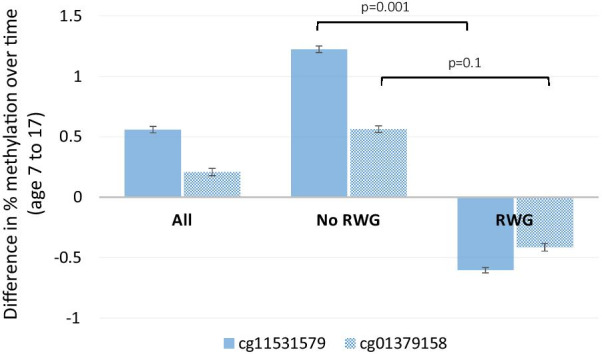
Change in methylation at the loci within individuals from age 7 to 17 by RWG. Test for differences using the Student’s t-test. Those who did not have RWG (*n* = 60) demonstrated small mean increases (cg01379158, + 0.6%; cg11531579, + 1.2%) in methylation, whereas those who had RWG (*n* = 34) demonstrated small (cg01379158, − 0.4%; cg11531579, − 0.6%) decreases in methylation between ages 7 and 17. Error bars represent standard deviation.

Overall, the two CpG sites which were positively associated with RWG tended to increase in methylation over time from childhood to adolescence within individuals (Fig. 4). However, when stratifying by RWG, in those who had RWG there was a decrease in methylation over time compared to those who did not experience RWG, particularly for cg11531579 (*p* < 0.001, Fig. 4).

### Childhood methylation in the replication cohort

Replication of the significant CpG sites was carried out using a similar UK-based cohort with data on growth in early life and epigenetic data in childhood (*n* = 104 at age 12). Compared to ALSPAC children, fewer experienced rapid growth in the first year in the SWS cohort; 29.7% had RWG (30/101) and 22.8% (23/101) had RT. Similar to the findings in ALSPAC, there was evidence of an association between RWG and DNA methylation at cg11531579 in the ISVA (*p* = 0.02) and SVA (*p* = 0.04) models, although the coefficients were smaller (0.005 and 0.004 in the ISVA and SVA models, respectively) (Table 3). There was no association for the either the cg01379158 site or the RT models.

### Genomic location of the differentially methylated CpG sites

The CpG site: cg01379158 was located upstream of the transcriptional start site in a CpG island (chr17:17,206,527–17,207,306). The nearest gene to cg01379158 is *NT5M*, also known as 5′,3′-Nucleotidase, Mitochondrial. The second differentially methylated CpG site (cg11531579, p_FDR_ < 0.05, Table 2) is located within a CpG island on chromosome 12: upstream 30+ kilobases is the protein coding gene Checkpoint With Forkhead And Ring Finger Domains (*CHFR*) (Table 2), whilst 558 base pairs downstream is a small (2 exons) non-coding region (AK055957), for which there is limited information.

## Discussion

### Summary

In this study, we identified that RWG in the first year of life was associated with small but significant increases in childhood DNAm (age 7) at two CpG sites (cg01379158 and cg11531579). The highest levels of methylation at the cg11531579 locus (age 7) were in those who had RWG and were either currently (age 7) or subsequently overweight/obese (age 17). Furthermore, there was suggestive evidence that this site was differentially methylated in the replication cohort. We did not find evidence of differentially methylated regions, of differential methylation associated with RT, or of differentially methylated BMI-associated candidate sites.

### Interpretation and comparison with previous findings

To our knowledge this is the first EWAS to identify differential DNAm associated with early life rapid weight gain. DNAm at the locus near *CHFR* (cg11531579) was higher in those who had RWG and who were overweight/obese in childhood or adolescence. Early life RWG was associated with small changes in childhood methylation, but not with methylation in adolescence, which could have been partly due to a smaller sample size or a lack of persistence in the differential DNAm seen in early life. Indeed, in those who had RWG there was a decrease in methylation over time (age 7 to 17), which may reflect the ‘recovery’ of hypermethylation in childhood. This phenomenon of attenuation of DNAm over time from signals detected in early life has been reported previously [24]. There is the possibility that methylation changes may be greater earlier in childhood closer to the timing of the exposure.

There are inherent links between birthweight and postnatal growth, and birthweight associated DNAm changes are also often related to growth control [40]. Rapid thrive accounts for catch-up growth from low birthweight, whereas RWG includes some of the effects of low birthweight. Although birthweight influences RWG, associations between RWG and adiposity remain after adjustment for birthweight [41]. As associations were stronger between DNAm (at the identified CpG sites) and RWG (rather than RT), it is plausible that methylation at these sites also encompasses some of the effects of catch-up growth from low birthweight.

Neither early life growth nor birthweight have been previously associated with DNAm at either of the identified sites. We searched the EWAS Catalogue (https://ewascatalog.org/) to assess whether any of the CpG sites had been previously identified in other EWAS, with results suggesting that both of these loci may be linked to bone composition and cholesterol metabolism, factors which could plausibly be linked to growth. As the DNAm changes identified were small, it is perhaps speculative to discuss the impact on gene expression.

The cg11531579 site, which was positively associated with RWG in ALSPAC and the SWS children, has nearby transcripts with cancer-associated roles [42–48]. The *CHFR* (cg11531579) gene encodes a E3 ubiquitin-protein ligase which regulates the cell cycle at the antephase checkpoint (prior to cell division) [42]. Differential epigenetic regulation of *CHFR* has been identified in cancer as a result of promoter hypermethylation [43, 44], or deacetylation of histones in the promoter region [45]; however it is unclear whether changes in expression are a cause or consequence of cancer. The CpG site is located within a DNAse I hypersensitivity cluster, which may suggest a transcription factor binding region, and a H3K27Ac histone mark, which is often found near regulatory elements and is thought to be a transcription enhancer, suggesting regulatory functions. Downstream of cg11531579 is AK055957, a small non-coding RNA regulatory sequence, with an uncharacterised biological role. Recently, this CpG (in combination with others) has been identified as a potential DNAm biomarker for use in detection panels for hepatocellular carcinoma [46] and pancreatic ductal adenocarcinoma [47], and is differentially methylated in children with acute myeloid leukaemia post-chemotherapy (− 0.24 change in beta value, p = 0.004) [48]. These findings suggest this locus may have a role in carcinogenesis, which may suggest a weak link with rapid growth.

The cg01379158 site is located in the transcriptional start site of the *NT5M* gene, a gene involved in nucleotide metabolism. The gene is located on chromosome 17 in the Smith-Magenis syndrome-critical region, which is a rare condition characterised by inverse circadian rhythm and disturbed sleep, factors which have also been linked to child obesity [49, 50]. At this CpG site there was no evidence of differential methylation in the SWS cohort, and methylation at the cg11531579 site was around half that observed in ALSPAC. There are several possible reasons for this, such as the moderate sample sizes, or smaller proportion of those who had rapid growth in the SWS. From the ALSPAC data it was evident that there is loss of methylation over time (age 7 to 17) at these loci in those with RWG; therefore as the SWS were also slightly older (+ 5 years) this may also explain less differential methylation. This may suggest that a biomarker for early rapid growth could have greater utility in early childhood and warrants further investigation in younger cohorts.

There were no associations between previously reported BMI-related CpG sites and RWG or RT arising from our candidate analysis, which could be for various reasons. First, the candidate loci mapped to genes with specific roles, which could be different to the mechanisms and pathways of RWG. Secondly, although some of the associations have been replicated in pre-school children [9], primarily the candidate loci were relevant to an adult population, whereas this cohort was sampled and analysed at a much younger age. Finally, RWG has been associated with subsequent changes in BMI, whereas Wahl and colleagues conclude that the majority of the identified BMI-related CpGs were a consequence (rather than a cause) of changes in BMI [12]. Thus, if rapid growth was associated with DNA changes in this subset of CpGs, this perhaps would have been more likely to have been as a consequence of changes in BMI. Indeed, current evidence suggests that the direction of the effect is from BMI to DNAm [12, 51]; therefore RWG (i.e. early life increases in BMI) associated DNAm changes may also be consequential of the phenotype rather than causal.

Similar to Reed et al*,.* [51], we did not identify strong direct associations between childhood methylation at these CpGs and later BMI. They did however identify associations between a DNAm score for BMI and health outcomes where BMI is a risk factor, suggesting methylation may have more utility as a biomarker of BMI-related morbidity than as a predictor of BMI itself [51].

### Strengths and limitations

A limitation of our study is the small sample size; however despite this, results were independently replicated at 1 of the CpGs. It will be important to replicate these findings in other cohorts with much larger sample sizes and a range of ages. This study has a number of strengths, principally the rare combination of detailed early life phenotypic and anthropometric data, as well as epigenetic data, as cohorts with longitudinal and epigenetic data of this nature are scarce. Other studies will undoubtedly also be limited by the lack of available early life weight measures, and future birth cohort studies should strive to collect these vital data. Whilst our analysis may have been underpowered, robust associations were still identified, although it is possible that other differentially methylated sites may have been missed.

Here we investigated RWG in the first year; however the entire childhood period could also be a critical period for growth and development of obesity [52]. There is the possibility that associations may be stronger in early life (before age 7), closer to the timing of the exposure, although this is not possible to test without multiple measures of DNAm throughout childhood.

There were differences in the associations identified in the models with and without adjustment for cell types. Whole blood represents a mixed cell population with varying proportions of white blood cells. Phenotypic variation in cell-type composition could confound analyses, or it could also represent an important physiological change in response to an exposure or disease, which may be related to the phenotype of interest. Obesity is an acknowledged chronic, inflammatory condition, and has been associated with inflammatory indicators including C-reactive protein [53] and white blood cell counts [54, 55]. When investigating biomarkers (related to an exposure) that are associated with an inflammatory disease outcome, to remove variation from cell counts could potentially disregard important loci.

We utilised SVA and ISVA to remove unwanted variation from confounders (which cannot always be adequately corrected for), whilst retaining differences due to the exposure of interest [56]. SVA finds sources of variation from the methylation data itself, and models these as linearly uncorrelated singular vectors (surrogate variables) which are then included as covariates in the regression model [33]. ISVA is a modified version of SVA where surrogate variables are deemed independent. In support of ISVA, known confounding factors such as age and batch are linearly uncorrelated statistically independent variables, therefore it would be appropriate to model these as independent variables. ISVA was shown to perform best at capturing a known specific biological signature when compared to other adjustment methods [34], although this may not hold true for all datasets [34, 57]. A thorough simulation study compared each of the common adjustment methods (Houseman’s reference-based method, RefFreeEWAS, SVA, ISVA, EWASher and RUV) and found no method performed perfectly for all parameters, but concluded SVA was most robust (and ‘safest’) [58]. In summary, as high-performing adjustment methods both were utilised in these analyses.

## Conclusion

Our findings suggest that differential DNAm at 2 loci could be markers of early weight gain. At one CpG site, the highest levels of childhood methylation were in those who had RWG in early life and were subsequently overweight/obese in childhood or adolescence; therefore this site may have use as a biomarker of subsequent overweight/obesity in those who experience RWG. The EWAS identified 2 potentially important candidate sites, which could be the focus of further investigation. Further work is required to determine whether these CpG sites are consistently, differentially methylated in different populations, time points, and ages.

## Supplementary information

**Additional file 1.** Southampton's Women’s Survey supplementary methods.

**Additional file 2.** Supplementary tables and figures.

**Additional file 3.** Top CpG hits by model for DNA methylation at ages 7 and 17.
