# Supplementary material for: Childhood DNA methylation as a marker of early life rapid weight gain and subsequent overweight
Source: Clin Epigenetics. 2021 Jan 12;13:8. doi: 10.1186/s13148-020-00952-z (PMC7805168; doi:10.1186/s13148-020-00952-z)
Supplement: Supplementary file 1 — Additional file 1. Southampton's Women’s Survey supplementary methods. [file 13148_2020_952_MOESM1_ESM.docx]

## Supplementary text

**SWS Cohort**

The Southampton Women’s Survey (SWS) is an ongoing, prospective cohort study of 12,583, initially non-pregnant, women aged 20–34 years, living in the city of Southampton, UK (39). Assessments of lifestyle, diet and anthropometry were performed at study entry (April 1998–December 2002). Women who subsequently became pregnant were followed through pregnancy and their offspring through infancy and childhood. Follow-up of the children and sample collection/analysis was carried out under Institutional Review Board approval (Southampton and South West Hampshire Research Ethics Committee, references 276/97, 307/97, 153/99w, and 10/H0504/30) with written informed consent. Here we focus on the follow-up of the children aged 11-13 years which included epigenetic data.

**Early life measures**

Birth weights were recorded by midwives attending the birth and weight at 12 months using hospital digital scales (Seca Ltd, London) that were regularly calibrated. In order to adjust for sex and gestational age and also to compare with reference values for the population, birth weight measurements were expressed as z-scores compared with the 1990 British Growth Foundation (CGF) data, and were used to determine the rapid weight gain and rapid thrive variables. Rapid thrive was determined as z-score_12m_ − r × z-score_birth_, where r was the cohort regression coefficient (r=0.276) of the linear model with z-score at 12month as the outcome with birthweight z-score as the exposure. RWG and RT were analysed as categorical variables of a >+0.67 standard deviation change.

**DNA extraction**

Genomic DNA (gDNA) was extracted from whole blood samples using the QIAamp Blood DNA mini kit (Qiagen). Quality of the genomic DNA was assessed by agarose gel electrophoresis and quantity of gDNA was checked on the NanoDrop ND-1000 (NanoDrop Technologies).

**Infinium Human MethylationEPIC BeadChip array**

DNA methylation using the Infinium Human MethylationEPIC BeadChip array was used to interrogate DNA methylation in 107 whole blood samples. 1µg of the genomic DNA was treated with sodium bisulfite using Zymo EZ DNA Methylation-Gold kit (ZymoResearch, Irvine, California, USA, D5007) and processing of the Human MethylationEPIC (Infinium Methylation EPIC; Illumina, Inc. CA, USA) platform was carried out by the Centre for Molecular Medicine and Therapeutics (CMMT) (http://www.cmmt.ubc.ca). The idat files were processed in R v3.5.2. Estimation of white blood cell counts was done using the Houseman algorithm. CpGs with a high detection p-value (p>0.01), beadcount<3, cross-reactive and polymorphic probes identified by Pidsley et al., (59) and probes on sex chromosomes were removed prior to downstream analysis (final number of CpGs=792,718). However, only CpG sites that were differentially methylated (p_FDR_<0.05) in the ALSPAC analysis were examined in the SWS samples.
