# Supplementary material for: Childhood DNA methylation as a marker of early life rapid weight gain and subsequent overweight
Source: Clin Epigenetics. 2021 Jan 12;13:8. doi: 10.1186/s13148-020-00952-z (PMC7805168; doi:10.1186/s13148-020-00952-z)
Supplement: Supplementary file 2 — Additional file 2. Supplementary tables and figures. [file 13148_2020_952_MOESM2_ESM.docx]

## Supplementary

Table 1 Body composition of ARIES participants age 7 and 17

|  | Age 7 | |  | Age 17 | |
| --- | --- | --- | --- | --- | --- |
|  | n | Mean (SD) |  | n | Mean (SD) |
| BMIz | 870 | 0.15(1.03) |  | 749 | 0.36(1.12) |
|  | n | % |  | n | % |
| Healthy weight | 753 | 87.25 |  | 597 | 80.68 |
| Overweight/obese | 110 | 12.75 |  | 143 | 19.32 |
| Total | 863 |  |  | 740 |  |

Proportion (%) of study members in healthy weight or overweight/obese and mean BMIz and standard deviation (SD). n, sample size; %, column percentage.

Table 2 Linear associations between CpG sites (age 7) and RT.

| **Exposure** | **n** | **CpG name** | **Chr** | **Nearest gene** | **Model** | **Coefficient** | **SE** | **p value** |
| --- | --- | --- | --- | --- | --- | --- | --- | --- |
| **With cell counts** | |  |  |  |  |  |  |  |
| RT | 116 | cg01379158 | 17 | *NT5M* | SVA | 0.0078 | 0.0025 | **0.0022** |
| **Without cell counts** | |  |  |  |  |  |  |  |
| RT | 125 | cg01379158 | 12 | *NT5M* | SVA | 0.0065 | 0.0025 | **0.0107** |
| RT | 125 | cg11531579 | 12 | *CHFR* | SVA | 0.0056 | 0.0028 | **0.0493** |

Models are adjusted for age, sex, and surrogate variables, both with or without adjustment for cell counts. Estimates represent beta coefficients. n, sample size; SE, standard error; SVA, surrogate variable analysis.

Table 3 Summary statistics of CpG methylation (age 7) at the identified differentially methylated loci (age 7) by phenotype (healthy weight not healthy weight) at ages 7 and 17

|  |  | N | Mean | SD | Median | Min | Max |  | N | Mean | SD | Median | Min | Max |
| --- | --- | --- | --- | --- | --- | --- | --- | --- | --- | --- | --- | --- | --- | --- |
|  | Age 7 | cg01379158 | | | | | |  | Age 7 | cg11531579 | | | | |
| No RWG | Healthy weight | 74 | 0.069 | 0.017 | 0.068 | 0.033 | 0.125 |  | 74 | 0.035 | 0.011 | 0.033 | 0.016 | 0.084 |
|  | OWOB | 10 | 0.075 | 0.021 | 0.071 | 0.042 | 0.106 |  | 10 | 0.039 | 0.011 | 0.041 | 0.024 | 0.054 |
| RWG | Healthy weight | 35 | 0.078 | 0.025 | 0.076 | 0.033 | 0.149 |  | 35 | 0.045 | 0.02 | 0.039 | 0.02 | 0.118 |
|  | **OWOB** | **6** | **0.091** | **0.019** | **0.09** | **0.064** | **0.114** |  | **6** | **0.057** | **0.013** | **0.059** | **0.042** | **0.075** |
|  | Total | 125 | 0.073 | 0.021 | 0.073 | 0.033 | 0.149 |  | 125 | 0.04 | 0.015 | 0.035 | 0.016 | 0.118 |
|  | p value | 0.017 |  |  |  |  |  |  | < 0.001 |  |  |  |  |  |
|  | Age 17 | cg01379158 | | | | | |  | Age 17 | cg11531579 | | | | |
| No RWG | Healthy weight | 54 | 0.069 | 0.017 | 0.069 | 0.039 | 0.103 |  | 54 | 0.036 | 0.012 | 0.033 | 0.023 | 0.084 |
|  | OWOB | 19 | 0.068 | 0.018 | 0.067 | 0.033 | 0.106 |  | 19 | 0.036 | 0.01 | 0.035 | 0.016 | 0.054 |
| RWG | Healthy weight | 28 | 0.076 | 0.026 | 0.074 | 0.033 | 0.149 |  | 28 | 0.041 | 0.014 | 0.037 | 0.02 | 0.086 |
|  | **OWOB** | **9** | **0.085** | **0.021** | **0.08** | **0.056** | **0.118** |  | **9** | **0.059** | **0.027** | **0.057** | **0.025** | **0.118** |
|  | Total | 110 | 0.072 | 0.02 | 0.071 | 0.033 | 0.149 |  | 110 | 0.039 | 0.015 | 0.035 | 0.016 | 0.118 |
|  | p value | 0.0801 |  |  |  |  |  |  | < 0.001 |  |  |  |  |  |

Values represent beta values. RWG, rapid weight gain; n, total in each group; SD, standard deviation; min, minimum; max, maximum. P value from ANOVA. Bold represents the high-risk phenotype of RWG in infancy and subsequent OWOB, which has higher DNAm levels (relative to the other categories).

| A. 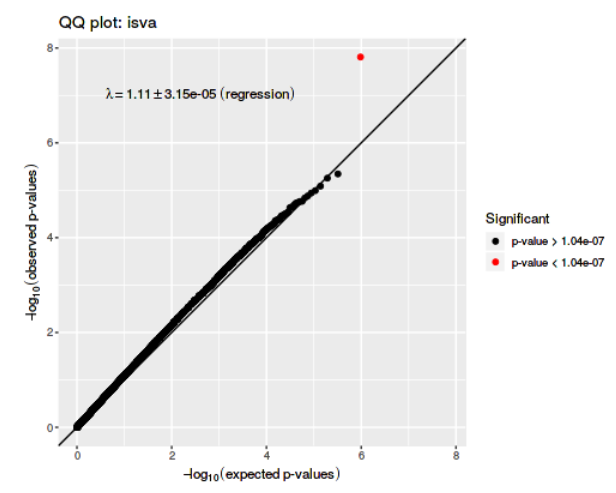 | B. 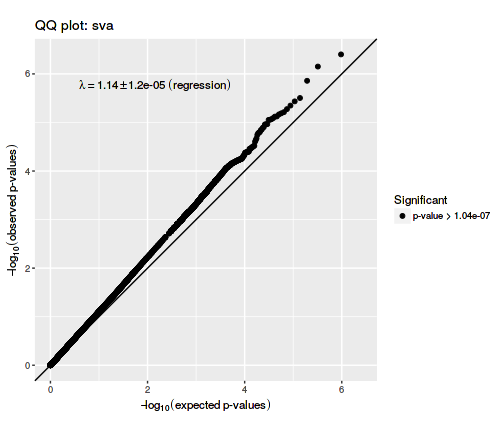 |
| --- | --- |
| C.  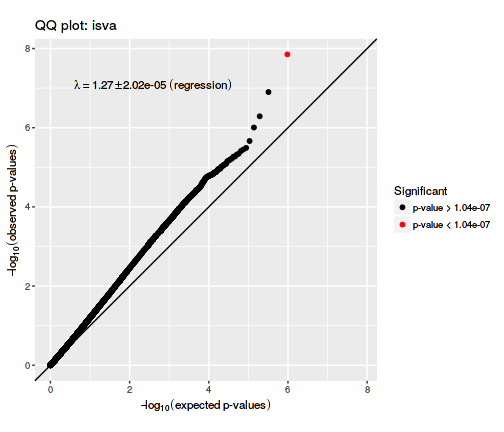 | D. 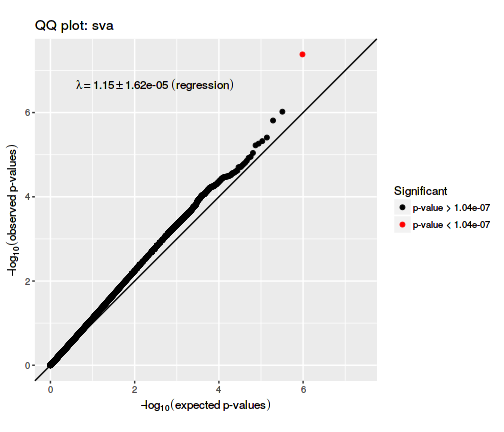 |

*Figure I Q-Q plots for RWG models (SVA, ISVA) both with (A, B) and without (C,D) adjustment for cell counts.*

*The Q-Q plots present the distribution of the p value for the association between CpG site methylation and RWG. The straight line is the expected distribution under the null hypothesis.*

| **Age 7, RWG** |
| --- |
| **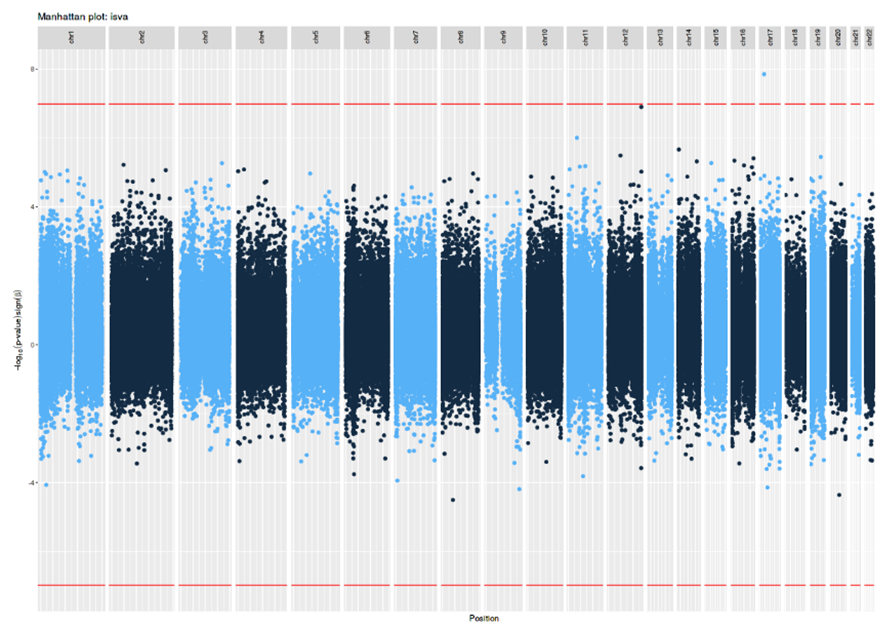**  **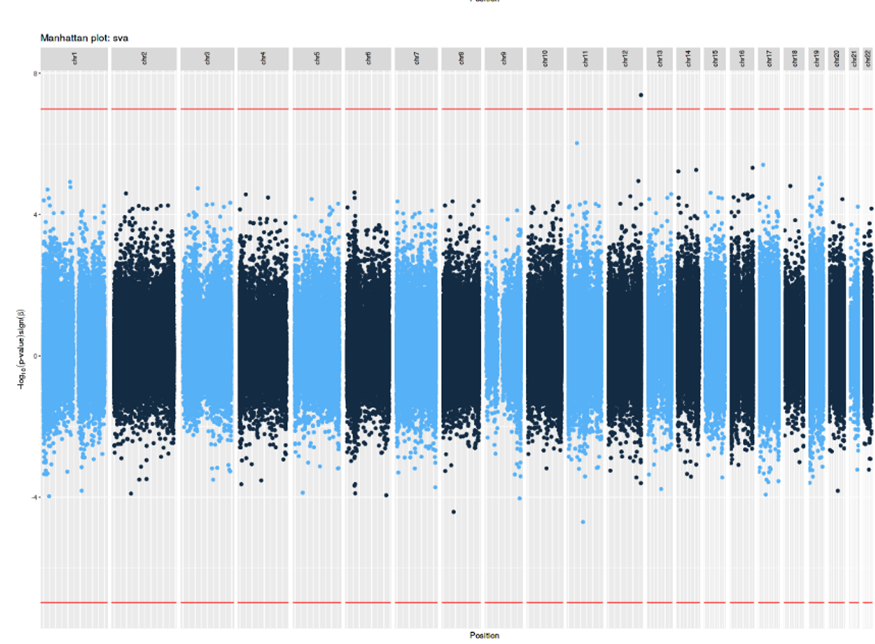** |
| **Age 7, RT** |
| **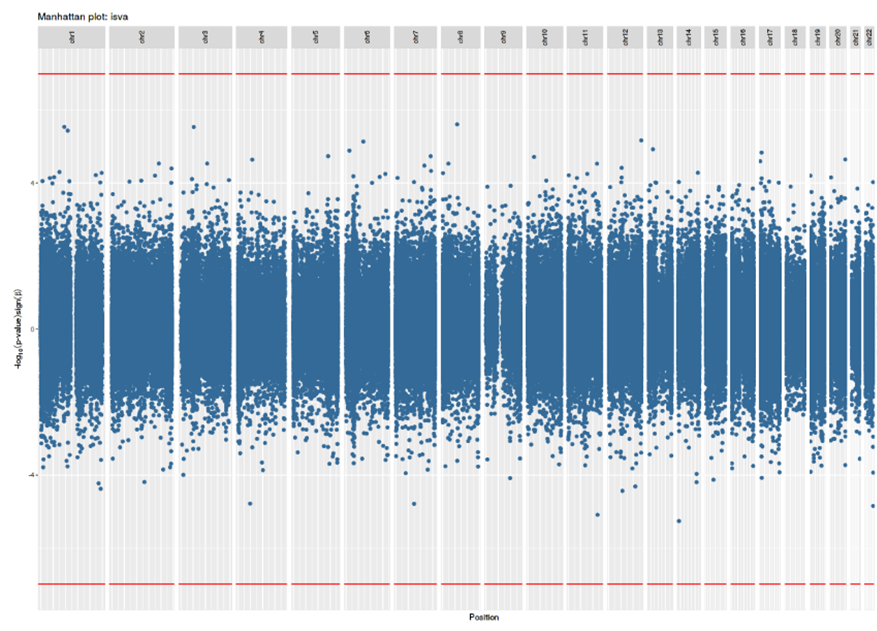** |
| **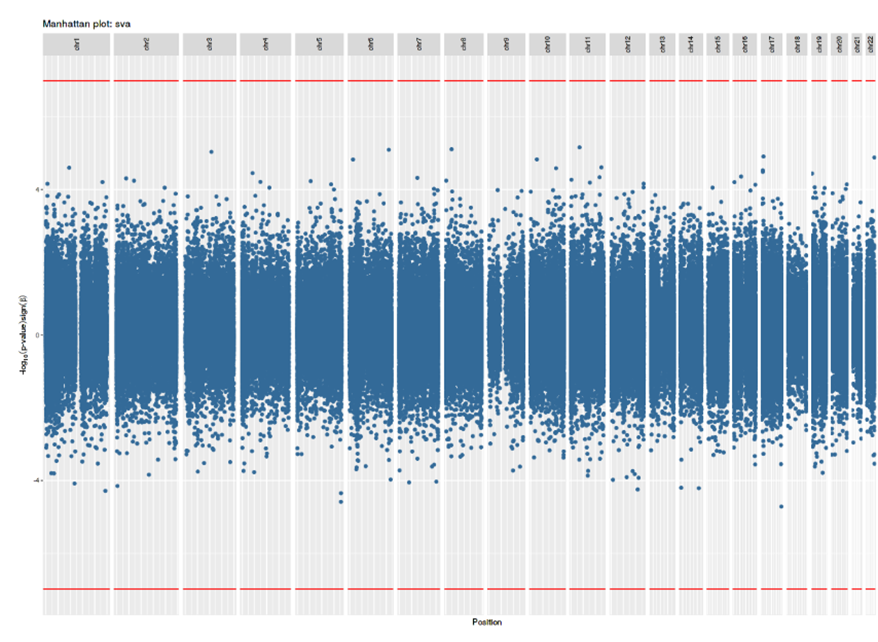** |
| **Figure 2 Bi-directional Manhattan plots of the EWAS analysis of RWG and RT for the ISVA and SVA models without cell counts.** The x-axis represents the chromosomes and the y-axis shows the –log10(P). The red line indicates the Bonferroni-corrected epigenome-wide threshold (p<1.04x10^-7^). Positively associated loci are displayed in the positive y-axis and negatively associated loci are displayed in the negative y-axis. |
